# PAQR3在肺癌中的表达及其临床意义

**DOI:** 10.3779/j.issn.1009-3419.2017.04.06

**Published:** 2017-04-20

**Authors:** 晓辉 梁, 保存 孙, 继媛 韩, 秀兰 赵, 增辉 刘

**Affiliations:** 300070 天津，天津医科大学病理教研室 Department of Pathology, Tianjin Medical University, Tianjin 300070, China

**Keywords:** PAQR3, 肺肿瘤, 表达, 临床意义, Progesterone and adiponectin receptor family 3, Lung neoplasms, Expression, Clinical significance

## Abstract

**背景与目的:**

孕酮及脂联素受体家族成员3（progesterone and adiponectin receptor family member 3, *PAQR3*）是最新研究发现的抑癌基因，通过抑制细胞增殖、细胞恶性转化、抑制血管生成及肿瘤转移来影响肿瘤的发展。本研究旨在探讨PAQR3在肺癌组织中的表达及其临床意义。

**方法:**

选取天津医科大学总医院病理科2008年1月-2010年12月经病理明确诊断的106例肺癌组织标本作为实验组，同时取其癌旁正常组织作为对照。所有患者的诊断均经术后病理诊断证实，采用免疫组织化学方法检测PAQR3蛋白在肺癌组织和癌旁正常组织中的表达，并探讨其表达的临床意义。

**结果:**

PAQR3蛋白在肺癌组织中阳性表达率显著低于癌旁正常组织（*P* < 0.01）；PAQR3蛋白的阳性表达率与患者的性别、年龄、肿瘤大小无关，与患者的病理分型、分化程度、TNM分期和有无淋巴结转移具有相关性（*P* < 0.05）；*Kaplan-Meier*生存分析结果显示PAQR3蛋白阳性表达的患者5年生存率高于阴性表达的患者（*P* < 0.05）。

**结论:**

PAQR3蛋白的表达量在肺癌组织中明显降低，提示PAQR3可能在肺癌的发病机制中发挥重要的作用。

## Abstract (en)

**Background and objective:**

Progesterone and adiponectin receptor family member 3 (PAQR3) is a recently discovered tumor suppressor gene, which affects the development of a tumor by inhibiting cell proliferation, cell malignant transformation, angiogenesis, and tumor metastasis. This study investigates the expression of PAQR3 in lung cancer and its clinical significance.

**Methods:**

A total of 106 patients with lung cancer received surgical treatment in hospital, and adjacent normal tissues of these patients were utilized as control group. The diagnosis of all patients was confirmed through clinical pathology. The expression of PAQR3 protein was detected by immunohistochemistry in lung cancer and adjacent normal tissues. The clinical significance of its expression was also investigated.

**Results:**

The positive expression rate of PAQR3 protein in lung cancer was lower than that in adjacent normal tissues (*P* < 0.01). The positive expression rate of PAQR3 protein was unrelated to age, tumor size, and gender, but it exhibited a significant relationship with the pathological type and differentiation, TNM staging, and lymph node metastasis (*P* < 0.05). *Kaplan-Meier* survival analysis showed that the five-year survival rate of patients with PAQR3 protein positive expression was higher than that in patients with negative expression (*P*=0.026).

**Conclusion:**

The expression of PAQR3 protein significantly decreased in lung cancer, indicating that PAQR3 protein plays an important role in the pathogenesis of PAQR3 in lung cancer.

肺癌是目前世界上发病率和致死率第一位的恶性肿瘤，也是我国境内最常见的恶性肿瘤之一。据国家癌症中心发布的数据显示，2009年至2011年我国肺癌患病率是733.3（1/10万）。其中男性509.3（1/10万），居恶性肿瘤的第1位。女性224（1/10万），居恶性肿瘤第2位^[1]^。而有57%的患者在初诊时已经发生了远处转移，导致晚期肺癌患者死亡的主要原因是侵袭和转移^[2, 3]^，因此深入了解肺癌的发生、发展和侵袭机制，研究新型靶向治疗药物，抑制肺癌的侵袭和转移，对于提高治疗效果、延长患者生存期尤其重要。新近研究发现孕酮及脂联素受体家族成员3（progesterone and adiponectin receptor family member 3, PAQR3）隶属于*PAQR*基因家族，是一种位于高尔基体上的七次跨膜蛋白。目前已有多项研究^[4-7]^证实PAQR3在多种恶性肿瘤中如乳腺癌、结直肠癌、胃癌、肝细胞癌、肾癌膀胱癌和骨肉瘤组织中发挥显著的抑癌作用。PAQR3可以通过抑制细胞增殖、细胞恶性转化、抑制血管生成及肿瘤转移来影响肿瘤的发展^[8]^。研读国内外文献，有关PAQR3蛋白在肺癌组织，尤其是人的肺癌组织标本中的抑癌作用研究仍为空白。本研究即采用免疫组织化学染色的方法对肺癌组织中PAQR3蛋白的表达情况进行了检测和分析，以进一步明确PAQR3与肺癌的病理特征及其预后的相关性，为优化肺癌的临床诊治方案提供新思路。

## 1 资料与方法

### 1.1 一般资料

收集天津医科大学总医院病理科2008年1月-2010年12月经手术切除、临床资料完整的肺癌病例106例，所有病例截止术前均未接受化疗或放疗，且无其他恶性肿瘤疾病，无严重肝、肾功能损害。106例肺癌组织标本均经HE染色，病理诊断明确。其中男性60例，女性46例，年龄30岁-70岁，平均年龄（47.5±6.4）岁；按照世界卫生组织（World Health Organization, WHO）（2015）肺肿瘤组织学分类，其中鳞癌37例，腺癌45例，小细胞癌15例，大细胞癌9例；肿瘤-淋巴结-转移（tumor-node-metastasis, TNM）分期，Ⅰ期20例，Ⅱ期32例，Ⅲ期31例，Ⅳ期23例；按组织学分级（不含腺癌），高分化10例，中分化16例，低分化35例（包括未分化）。其中伴有淋巴结转移66例，未转移40例。同时取各肺癌病例的癌旁正常组织作为对照。随访资料截止到2015年12月，8例患者失访，随访率92.4%。1年、3年和5年生存率分别为81%、51%和42%。

### 1.2 免疫组织化学染色试剂与方法

常规使用中性福尔马林固定，经梯度酒精脱水、二甲苯透明、浸蜡，石蜡包埋后常规切片，厚度4 μm。放入65 oC烤箱2 h，之后进行二甲苯脱蜡和梯度酒精水化，滴加抗原修复液进行修复抗原，后使用封闭液室温封闭1 h，加入浓度为1:50的PAQR3抗体稀释液（美国Santa Cruz公司），在恒温培养箱中孵育2 h（37 oC）。使用PBS冲洗并加入二抗稀释液（美国Santa Cruz公司），在恒温培养箱中孵育1 h（37 oC）。之后使用PBS冲洗并加入辣根过氧化物酶标记的链霉卵白素，采用DAB显色，苏术精复染，显微镜下观察。

### 1.3 免疫组织化学染色结果判定

所有染色标本由两位病理诊断医生在双盲的情况下进行免疫组织化学染色的结果判定。判定标准：以细胞浆中出现棕黄色颗粒为阳性细胞。按照阳性细胞率和染色强度两个分级做为判定标准。以细胞浆染色强度分别计分：无着色为0分，淡黄染色为1分，棕黄色为2分，棕褐色/棕黑色颗粒为3分；以细胞阳性范围分别计分： < 5%为0分，5%-25%为1分，26%-50%为2分，51%-75%为3分，>75%为4分；再将染色强度与阳性细胞率作乘积分级：>3分为（+），6分-9分为（++），9分-12分为（+++），凡（+）或（+）以上者均为阳性表达。

### 1.4 统计学方法

应用SPSS 20.0统计软件进行数据分析，计数资料对比采用*χ*^2^检验，生存情况分析采用*Kaplan-Meier*法，以*P* < 0.05为差异有统计学意义。

## 2 结果

### 2.1 PAQR3蛋白在肺癌组织和正常肺组织中的表达

本研究中所有肺癌的诊断均经过术后HE染色结果证实，免疫组织化学染色（图 1）结果显示PAQR3主要在细胞浆中表达，呈褐色。PAQR3在正常组织中阳性表达率为71%，呈现高表达，在肺癌组织中阳性表达率为35%，表达水平明显较低。两组表达的差异具有统计学意义（*P* < 0.01）（表 1）。

**1:**
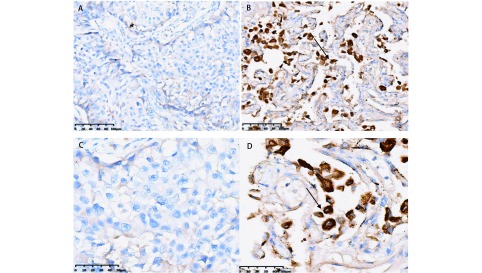
肺癌和癌旁正常肺组织中PAQR3的表达（免疫组化染色，A、B：200×，C、D：100×）。A、C：肺癌组织中PAQR3的阴性表达；B、D：正常肺组织中PAQR3的阳性表达（箭头所示） The expression of PAQR3 in lung cancer and adjacent normal tissues (IHC, A, B: 200×; C, D: 100×). A, C: Negative expression of PAQR3 in lung cancer tissue; B, D: Positive expression of PAQR3 in adjacent normal tissues

**1:** 肺癌组织和癌旁正常组织中PAQR3阳性表达结果分析 The expression of PAQR3 in the lung cancer and normal tissue

Group	PAQR3 expression		*P*
	Positive [*n* (%)]	Negative [*n* (%)]	
Lung cancer	35 (33.0)	71 (67.0)	< 0.01
Normal tissue	75 (70.7)	31 (29.2)	

### 2.2 PAQR3的表达与肺癌临床病理特征间的关系

肺癌患者PAQR3蛋白的阳性率在TNM分期、有无淋巴结转移、病理分型以及分化程度方面差异具有统计学意义(*P* < 0.05), 而年龄、性别、肿瘤大小的分布方面差异无统计学意义(*P*>0.05)(表 2).

**2:** PAQR3的表达与肺癌临床病理特征之间的关系 Expression of PAQR3 in lung cancer and its relationship with several clinical pathological parameters

Factors	*n*	PAQR3		*P*
		Positive	Negative	
Gender				> 0.05
Male	60	21	39	
Female	46	14	32	
Age (yr)				> 0.05
< 60	57	21	36	
≥60	49	14	35	
Tumor size (cm)				> 0.05
< 3	46	15	31	
≥3	60	20	40	
Pathological type				< 0.05
SCC	37	14	23	
Adenocarcinoma	45	19	26	
SCLC	15	2	13	
Large cell cancer	9	0	9	
TNM stage				< 0.05
Ⅰ	20	12	8	
Ⅱ	32	11	21	
Ⅲ	31	8	23	
Ⅳ	23	4	19	
Differentiation				< 0.05
Poor	35	8	27	
Intermediate	16	7	9	
Well	10	5	5	
Metastasis				< 0.05
Yes	66	14	52	
No	40	21	19	
SCC: squamous cell carcinoma; SCLC: small cell lung cancer; TNM: tumor-node-metastasis.				

### 2.3 PAQR3的表达与临床预后之间的关系

*Kaplan-Meier*生存分析显示，PAQR3阴性患者的5年中位生存时间为28.5个月，阳性患者的中位生存时间为45个月；PAQR3阳性表达者5年生存率明显高于阴性表达者，差异有统计学意义（*P* < 0.05）（图 2）。

**2:**
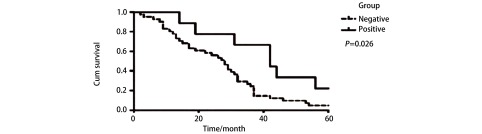
PAQR3表达量与肺癌患者5年生存率之间的关系 *Kaplan-Meier* survival curve of the expression of PAQR3 in 106 patients with lung cancer

## 3 讨论

肺癌是严重危害人类生命和健康的一种最为常见的恶性肿瘤，发病率和死亡率居高不下。近年来，随着细胞生物学和分子生物学的不断发展，对恶性肿瘤尤其肺癌的发病、侵袭和转移机制的研究取得了不少的成果。但是肺癌的发病率和死亡率仍没有明显的降低。因此，探讨和研发新的诊疗措施势在必行。肺癌具有多种癌基因参与、多种抑癌基因发生变化、分阶段变化的独特的生物学行为特点，因此我们应该更多的从分子生物学角度去探讨发病机制和侵袭转移的机制，从而研发新的分子靶向治疗药物。

*PAQR3*基因隶属于PAQR家族，位于染色体4q21.21上，在大多数物种里编码同一种蛋白，具有高度保守性。PAQR3蛋白定位于高尔基体上，是一种具有 Ⅲ型拓扑结构的七次跨膜蛋白，其N端朝向细胞质一侧、C段朝向高尔基体腔内，负责传递和调控胞内信号的作用。由于其最初被发现可以将细胞内游离的Raf激酶锚定在高尔基体上，使其不能被上游分子激活并且失去活化下游分子MEK的能力，因此又被称为RKTG（Raf kinase trapping to Golgi）。在机体内的肝脏、肾脏、脑、乳腺和睾丸组织中均存在高表达^[9, 10]^。已有研究^[4-8]^证实PAQR3在结直肠癌中发挥了抑癌作用，除此之外也被证明在多种肿瘤组织如乳腺癌、胃癌、肝细胞癌、肾癌膀胱癌和骨肉瘤组织中表达降低。众所周知RAS/RAF/MEK/ERK信号通路在肿瘤组织中持续激活，负向调控此条信号通路可对肿瘤细胞的增殖和侵袭产生明显的抑制作用，同时也会加速肿瘤细胞凋亡^[11]^。PI3K/AKT信号通路同样在肿瘤组织中持续激活，抑制PI3K/AKT通路的活性可以显著抑制肿瘤的增殖和促进凋亡^[7]^。那么PAQR3抑癌的最重要的机制是其能够负性调控RAS/RAF/MEK/ERK信号通路和PI3K/AKT信号通路的活化^[12]^。另有研究^[13]^发现，过表达PAQR3可通过上调P27KIPI蛋白表达，降低Cyclin D1蛋白的表达，从而影响细胞的周期，导致肿瘤细胞进入S期的比例减少，处于G_0_期/G_1_期的比例增加。使用SiRNA干扰下调细胞内的PAQR3表达后，P27KIPI蛋白表达明显减少，Cyclin D1蛋白则显著增加。而研究证实过表达PAQR3还可以明显降低基质金属蛋白酶9（matrix metalloproteinase 9, MMP9）的表达，而MMP9可以特异性的降解细胞外基质，从而促进肿瘤细胞的转移和浸润中，这可能也是PAQR3抑制肺肿瘤细胞转移和浸润的机制之一^[14]^。PAQR3参与了肿瘤发生与发展过程中的多条信号通路的调节，在抑制肿瘤侵袭与转移的生物学行为中起到了重要的作用。因此，我们推测PAQR3可能在肺癌患者的肿瘤组织中也同样存在异常表达并对患者预后产生重要影响。因此深入分析PAQR3在肺癌组织中的表达对肿瘤的发生、侵袭和转移的影响，对临床诊疗及研发新的分子治疗靶点都具有重要意义。

本研究结果证实PAQR3在正常肺组织中的表达显著高于肺癌组织，提示PAQR3的正常表达在肺癌的发生发展过程中发生了重大改变。PAQR3的表达率与患者的性别、年龄以及肿瘤的大小没有相关性，但是与肺癌患者的病理分型以及分化程度具有显著的相关性，恶性度越高，分化程度越低，PAQR3的阳性表达率越低，提示PAQR3的表达与肿瘤的生物学行为有密切的关系，同时其在肺癌中的阳性表达率与患者的临床分期以及有无淋巴结转移具有显著相关性，中晚期和有淋巴结转移的患者阳性表达率显著降低，这强有力的证实PAQR3参与抑制了肺癌的增殖和侵袭转移。进一步生存分析发现，PAQR3的阳性表达率与患者的预后存在着显著相关性，PAQR3阳性表达者5年生存率明显高于阴性表达者，推测肺癌组织中PAQR3的表达可以作为评估预后的重要参考指标。在体外细胞实验和动物体内实验中，己经证实过表达PAQR3可以显著抑制肿瘤细胞的克隆形成、体外增殖及体内成瘤能力^[15]^。因此，结合以往研究和本实验结果，我们推测PAQR3在抑制肺癌的发生、发展和侵袭中发挥重要的作用。

总之，PAQR3在肺癌组织中的表达受到明显抑制，与肿瘤的生物学行为、临床分期及患者预后存间在显著相关性，在术后检测肺癌肿瘤组织标本中的PAQR3对于评估患者预后具有重要意义，另外，开发靶向作用于PAQR3的药物也可能会给肺癌的治疗带来新的思路和方法。
